# Ancestral Informative Marker Selection and Population Structure Visualization Using Sparse Laplacian Eigenfunctions

**DOI:** 10.1371/journal.pone.0013734

**Published:** 2010-11-04

**Authors:** Jun Zhang

**Affiliations:** Department of Radiology, The University of Chicago, Chicago, Illinois, United States of America; Erasmus University Medical Center, Netherlands

## Abstract

Identification of a small panel of population structure informative markers can reduce genotyping cost and is useful in various applications, such as ancestry inference in association mapping, forensics and evolutionary theory in population genetics. Traditional methods to ascertain ancestral informative markers usually require the prior knowledge of individual ancestry and have difficulty for admixed populations. Recently Principal Components Analysis (PCA) has been employed with success to select SNPs which are highly correlated with top significant principal components (PCs) without use of individual ancestral information. The approach is also applicable to admixed populations. Here we propose a novel approach based on our recent result on summarizing population structure by graph Laplacian eigenfunctions, which differs from PCA in that it is geometric and robust to outliers. Our approach also takes advantage of the priori sparseness of informative markers in the genome. Through simulation of a ring population and the real global population sample HGDP of 650K SNPs genotyped in 940 unrelated individuals, we validate the proposed algorithm at selecting most informative markers, a small fraction of which can recover the similar underlying population structure efficiently. Employing a standard Support Vector Machine (SVM) to predict individuals' continental memberships on HGDP dataset of seven continents, we demonstrate that the selected SNPs by our method are more informative but less redundant than those selected by PCA. Our algorithm is a promising tool in genome-wide association studies and population genetics, facilitating the selection of structure informative markers, efficient detection of population substructure and ancestral inference.

## Introduction

Understanding genetic structure of human population is of fundamental interest in many applications. In population genetics, it has been widely used for inference of population evolutionary histories. In medical genetics, spurious associations can arise in the presence of population substructure. Detection and correction of population structure is a necessary step in genome-wide association studies. With the availability of high-throughput genotyping data in genome-wide disease studies, there has been increased interest in population structure. Correctly quantifying and understanding the genetic variation of human population is a challenging task. PCA has been used as a dominant method to identify population structure in the literature [1]–[3]. As a classical statistical tool to achieve dimension reduction, principal components (PCs) are linear combinations of the underlying variables and usually several top PCs can explain a large amount of variation in the whole dataset. For population based case-control association studies, the confounding effect due to population stratification can be effectively counted for by including the top PCs as covariates in a regression setting [3]–[5].

Further identifying a small panel of structure-informative markers that can be used to unravel population structure is also desired, since it can achieve genotyping savings and provide insight to genetic regions that undergone the evolutionary forces. This topic has been extensively studied in the literature [6]–[9]. A MCMC based program STRUCTURE [6] has been widely used for assigning individuals to clusters of populations. However, the expensive computing cost becomes impractical for disease studies involving genome scale markers and thousands of individuals. The result is also sensitive to the prior assumption of the number of underlying subpopulations. Other existing approaches such as information theory based informativeness for assignment [7] 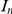, 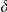 and 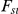 are allele frequencies based and require prior knowledge of individuals' ancestral memberships, which limits the application to admixed populations such as African Americans or individuals whose ancestral information is unknown. Recently Paschou et al. [10], [11] used the square sums of top PCs' entries as the weights to rank the informativeness of markers, which outperformed the approach of informativeness for assignment using statistic 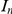 on worldwide human populations. Similar PCA based approaches have also been widely used to select a small set of PCA-correlated SNPs to correct population stratification [12]–[14].

However, PCA also has its limitation. It is sensitive to outliers which is caused by the fact that it actually computes the projection that maximizes the preservation of pairwise squared distances. The squaring of distances tends to preserve larger distances at the expense of preservation of short distances. The top PCs emphasize global patterns of the data, while the substructure of the data tends to appear in the lower ranked PCs. In the presence of outliers, pairwise distances involving outliers are significantly larger than other pairwise distances, which makes PCA tend to preserve the outlying structure rather than the bulk of the data. Also, the inclusion of extra PCs for population structure usually leads to power loss in association testing [15].

Motivated from geometric learning, new approaches [16]–[18] based on spectral graph theory [19] have been recently proposed to summarize population structure. Different from PCA, the methods use the idea of shrinkage and they preserve the local dependence structure of the study subjects. The proposed algorithms are nonlinear and robust to outliers, where one regards each subject as a vertice of a weighted graph [19] and makes edges only to its close neighbors, instead of all subjects in the study (see **Materials and Methods**). This reflects the fact that distances between vertices that are far apart are usually meaningless than closely correlated ones. The weight associated to edges for each pair of subjects measures their degree of being related. This adjacency graph approximates the underlying dependence structure of the sample population and the eigenvectors of the associated graph Laplacian contain useful geometric structure information (for details see the references above). The corresponding Laplacian eigenmap formed by embedding subjects to a lower dimensional Euclidean space via the top few eigenfunctions has locality preserving property. That is, distance between a pair of subjects in the embedded space reflect theirs degree of being correlated. The more they are correlated, the closer they are mapped to. Therefore Laplacian eigenmap clusters subjects who either come from the same discrete subpopulation or share more common ancestry from an admixed population and is ideal from revealing population structure.

Because of the limitation of PCA mentioned above, those PCA based approaches can be potentially problematic in the presence of outliers. In this paper, we use the global HGDP diversity panel to demonstrate that the markers selected based on Laplacian eigenfunctions in a regression setting (see **Materials and Methods**) are more informative but less redundant than the ones based on PCA approach (see examples below). Additionally, those most informative markers are typically sparse in the whole genome since they usually take only a very small percentage (less than 1%) of the total number of markers. Neither of the existing approaches in the literature has used this sparsity priori. Furthermore, we show that suitably incorporating the sparsity can significantly improve the overall performance on the HGDP panel. Therefore, we propose a sparse version of graph Laplacian eigenfunctions to select structure most informative markers which are also ancestry informative and can also be efficiently used to visualize the underlying population structure and correct population stratification in association studies. To compare the informativeness of selected SNPs with the PCA approach, we split the HGDP dataset equally into a training set and a testing set, and use the standard Support Vector Machine (SVM) [20]–[22] to predict the continental memberships of the samples (see **Results** for details). On the worldwide population HGDP panel, the proposed sparse Laplacian approach not only outperforms the PCA approach on the population membership prediction, the set of selected markers is strikingly less redundant than that by PCA. Therefore it is valuable for studies involving genome-wide biomarkers of thousands of individuals.

## Results

### Simulation study of a ring population

**Figure 1:**
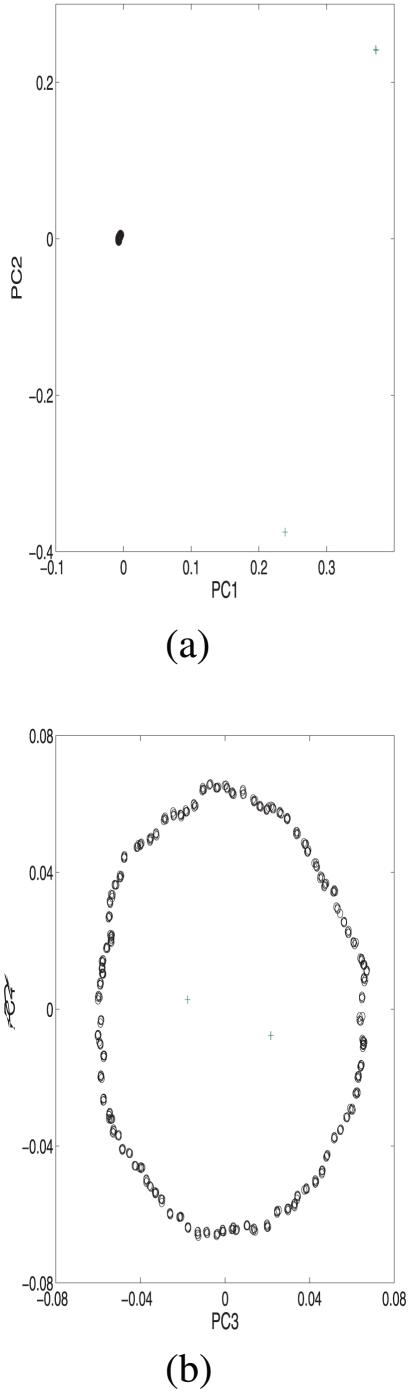
The top four PCs of a ring species and two outlier subpopulations. (a) shows that the PC1 and PC2 emphasize the two outliers; (b) PC3 and PC4 capture the underlying structure.

**Figure 2:**
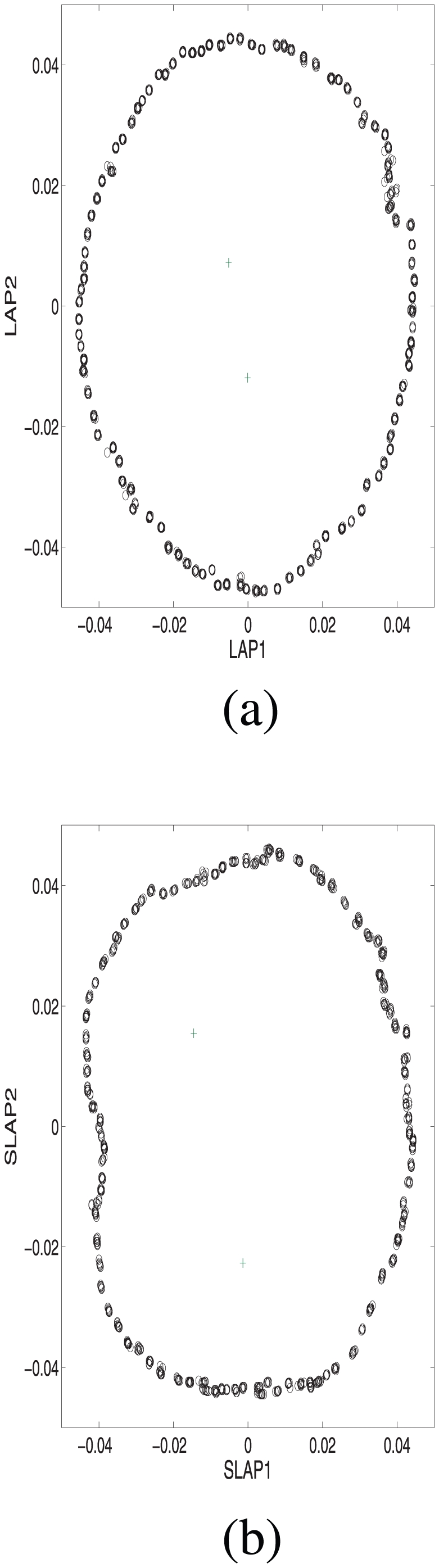
The top two Laplacian eigenfunctions of a ring species and two outlier subpopulations and its sparse version with only 300 most informative markers out of total 10,000 markers, where 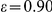.

We first applied PCA to the covariance matrix of this simulated sample. From **Figure 1**, one observes that the PC1 and PC2 distinguish the ring species from the two outlier subpopulations well, while the ring structure of the species, together with the two outliers, is detected by lower ranked PC3 and PC4. From **Figure 2**, one sees that the top two Laplacian eigenfunctions, LAP1 and LAP2, describe the ring structure and the two outlier subpopulations very well. Further comprehensive comparison of PCA and Laplacian eigenfunctions is available in the literature [16], [18], [23]. Next we used the marker selection procedure described below (see **Materials and Methods**) and selected top 300 informative markers out of total 10,000 markers. With these selected markers, the sparse Laplacian eigenfunctions, SLAP1 and SLAP2, recover a similar population structure without much information loss. Their correlation coefficients with the LAP1 and LAP2 are respectively 0.9912 and 0.9910. To measure the similarity of the two Figures 2(a) and 2(b), the Mantel test [24] based on pairwise distance is carried out with a highly significant Z-statistic value 2037.11.

### Global genomic variation of HGDP-CEPH dataset

**Figure 3:**
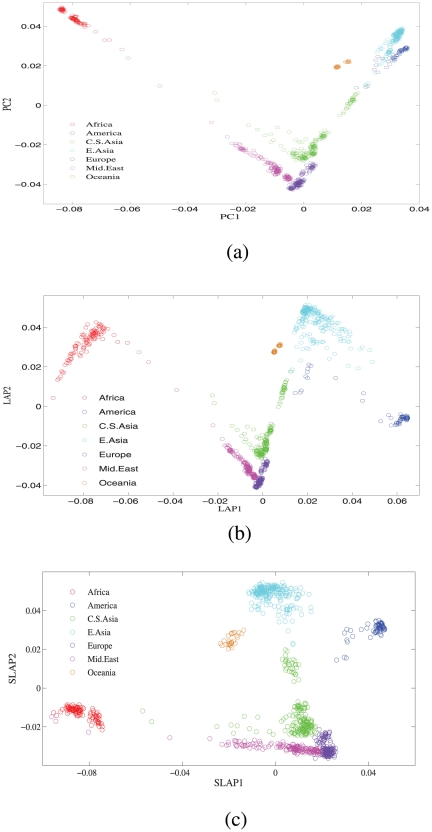
The global population structure of population sample HGDP-CEPH. Summarized by: (a) the top two Principal Components; (b) the top two Laplacian eigenfunctions using all available 647,483 SNPs; and (c) the top two Sparse Laplacian eigenvectors using the top 1,000 most informative SNPs. Here the parameter 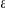 is set to be 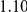.

After the preliminary data cleaning and normalization (see **Materials and Methods** for details) we first computed the standard top principal compents of the HGDP global sample. The biplot of PC1 and PC2 distinguishes the seven continents very well, except that there is some overlap of individuals from East Asia and America (see **Figure 3 (a)**). Next we computed the top Laplacian eigenfunctions with varying parameter 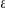. For large values of 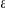, the biplot of LAP1 and LAP2 gives very similar global patterns observed in biplot of PC1 and PC2. Tuning the 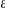 slightly, we can observe some fine local strucuture such as the structures of East Asia and America and their clear classification in **Figure 3 (b)**. Finally we applied the proposed algorithm to identify the most structure informative markers for the top 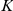 Laplacian eigenfunctions. Here 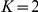 in the computation. The loading vectors 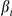
's are very sparse and have more than 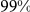 of the entries are vanishing. We computed the top two sparse Laplacia eigenfunctions, SLAP1 and SLAP2, using the selected top 1,400 SNPs. Their correlation coefficients with the LAP1 and LAP2 using all data are respectively 0.5275 and 0.5221. The Z-statistic of the Mantel test for the two Figures 3(b) and 3(c) is 1867.04. The biplot of SLAP1 and SLAP2 preserves the essential geographic patterns as observed in biplot of LAP1 and LAP2, see **Figure 3 (c)**. Even more, the clusters of C.S.Asia, E.Asia and America are slightly better separated.

### Intra-continent population structure

**Figure 4:**
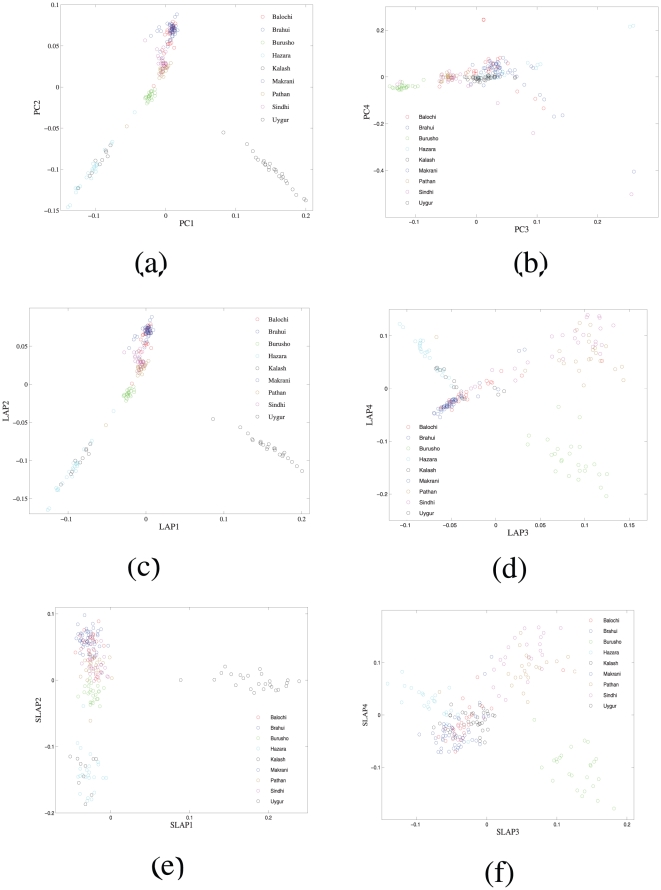
The population structure of Central and South Asia summarized by the top four principal compents and Laplacian eigenfunctions using all available 647,483 SNPs and its sparse version using only the top 747 most informative SNPs. Here the parameter 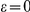.

We also explored the intra-continental structure in the HDGP-CEPH data using Laplacian eigenfunctions. Here we demonstrate it on the Central and South Asian population group consisting of total 207 individuals. The biplot of LAP1 and LAP2 gives almost identical gobal pattern as given by biplot of PC1 and PC2, see **Figure 4**. The biplot of PC3 and PC4 mainly identifies several outliers faraway from the clustering of the rest individuals. While with a suitably small 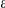, the biplot of LAP3 and LAP4 clearly distinguishes the Burusho subpopulation out. Next, we applied our algorithm to select the most informative SNPs for these top four Laplacian eigenfunctions. With the top 747 SNPs, we recovered the main population structure as the structure above obtained using all available SNPs. Their correlation coefficients with the top four Laplacian eigenfunctions using all data are respectively 0.9875, 0.9846, 0.9212 and 0.8998. The Z-statistics of the Mantel similarity test for the two pairs of Figures 4(c) and 4(e) and Figures 4(d) and 4(f) are respectively 409.37 and 404.98.

### Informative SNPs predicts continent membership via Support Vector Machine

**Figure 5:**
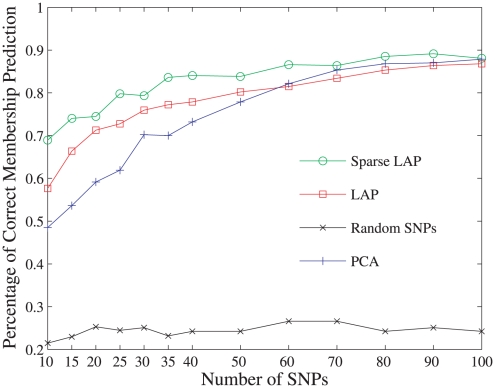
The performance of three approaches. Sparse Laplacian and Laplacian with top two eigenfunctions and PCA with top 18 PCs on the population memebership prediction, where the global population sample HGDP-CEPH were split into training and testing subsets with 470 individuals each.

To further validate the selected SNPs as signatures of population structure, we study the performance of predicting the continental memberships of the samples using the panel of most informative SNPs. We randomly split the total 940 individuals equally into a training set and a testing set. For individuals in the training set, the class labels are simply assigned to be 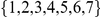 to stand for their corresponding seven continental memberships of Africa, Middle East, Europe, Central and South Asia, East Asia, Oceania and America. We use a standard SVM [20] to achieve our multi-class classification task with the top most informative SNPs. SVM is a supervised learning method which constructs a hyperplane or set of hyperplanes in a high-dimensional space typically for classification and regression tasks. Intuitively, a good separation is achieved by the hyperplane that has the largest distance to the nearest training datapoints of any class, since in general the larger the margin the lower the generalization error of the classifier. In all the experents carried out, the radial basis function is used as the default kernel function. The experiment is repeated 10 times and the average percentage of correct continental membership prediction is shown in **Fig 5**.

Here we also selected the informative markers using the PCA based approach [10], [11]. We reminder the readers that this approach transforms the genotype matrix 
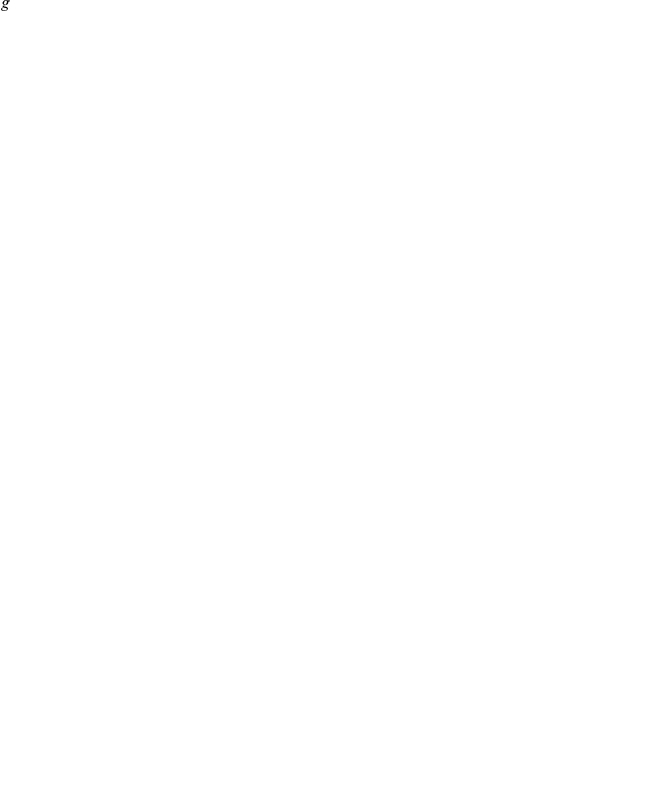
 differently from the standard normalization by setting the heterogenious genotypes to 0 and the homogenious wild/mild genotypes to +1/−1. Here we denote the updated data matrix as 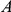. For optimal performance, we next estimated that the top 18 principal components of 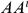 are significant, all of whose entries are then summed to select the ancestral informative markers. However, the initial identified set of informative markers by PCA is very redundant, see the summarized distribution of the linkage disequilibrium (LD) measure 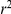 in Table 1. Finally we use the designed QR algorithm [11] to select the first 100 less correlated markers among the initial top 500 most informative markers identified by PCA.

**Table 1:** Summary of 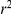 among the top 500 informative SNPs.

Rank	PCA	Lap	Slap
r2 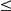 0.1	88422	118061	124457
0.1 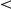 r2 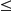 0.2	26604	6401	219
0.2 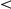 r2 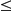 0.3	7141	260	31
0.3 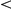 r2 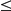 0.4	1342	6	15
0.4 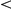 r2 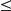 0.5	365	6	11
0.5 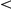 r2 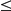 0.6	193	4	3
0.6 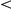 r2 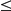 0.7	195	4	6
0.7 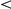 r2 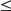 0.8	179	1	1
0.8 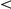 r2 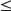 0.9	140	3	5
0.9 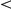 r2	169	4	2

Distribution of numbers of pairs among the most informative 500 SNPs identified by PCA without redundancy removed, Laplacian and Sparse Laplacian approachs for seven global continental population structure.

From the results in **Fig 5**, we can see first that the PCA approach is effective as compared with the poor result predicted by random SNPs. Next, with only the top two eigenfunctions the Laplacian approach (LAP) without sparsity consideration, which is equivalent to setting the penalty parameters 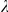
's to zero in the general framework (see **Materials and Methods**), is comparable with the PCA approach using all 18 significant PCs and redundancy removal procedure on prediction performance. Finally as expected, the sparse Laplacian approach (SLAP) improves the performance uniformly and works the best.

**Figure 6:**
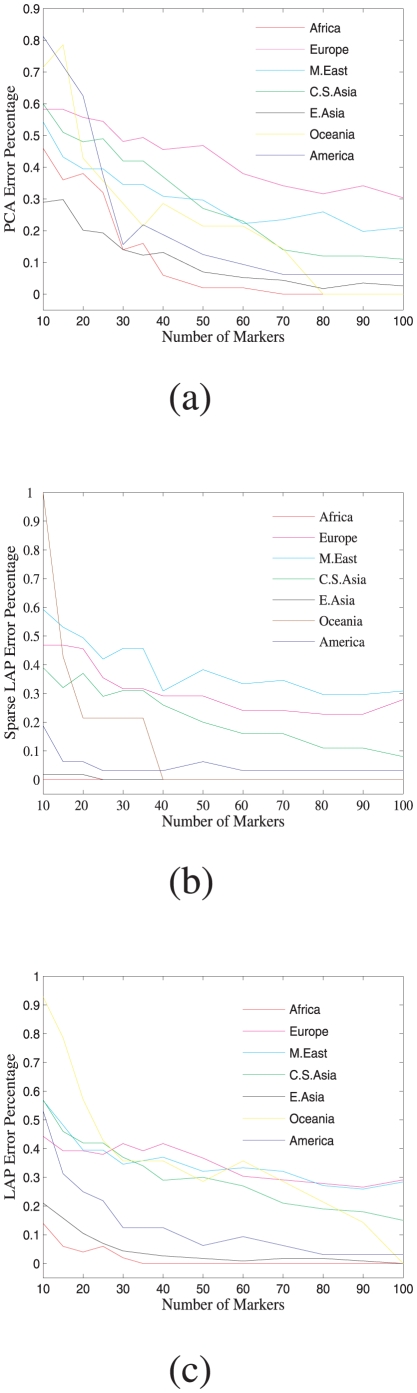
Prediction error percentages of PCA, Sparse LAP and LAP approaches of assigning individuals to their continental memberships.

**Figure 7:**
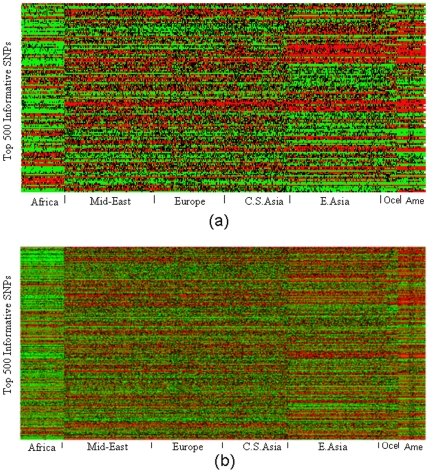
Comparison of the top informative markers. The homogeneous genotypes of wild alleles indicated with green, homegeneous genotype of mild alleles indicated with red, the heterogeneous genotype indicated with black and the missing genotypes indicated with yellow. (a) the top 500 ancestral informative SNPs identified by sparse Laplacian approach; (b) the top 500 ancestral informative SNPs identified by PCA approach.

The error percentage of assigning individuals to their populations of the three approaches is also provided in **Fig 6**. There, for example, one can observe that for Americans Laplacian approach has reduced prediction error than PCA and sparse Laplacian has even no prediction errors. The top 500 informative SNPs identified by the proposed sparse Laplacian eigenfunction approach and PCA are both shown in **Fig 7**. Interestingly, the SNPs of Africa are dominantly green(wild alleles), and the PCA identified SNPs are dominantly red(mild alleles) for the three continents of East Asia, Oceania and America. However, this homogeneity of the alleles for the three continents makes it difficult to distinguish the continent memberships among them. For example, in the PCA experiments of **Fig 5** quite a few individuals from American were mistakenly predicted as from East Asia. This is partly due to the clustering of America and East Asia in the biplot of the top principal components, which was observed earlier. While that is a relatively easy task using the SNPs identified by sparse Laplacian approach.

**Figure 8:**
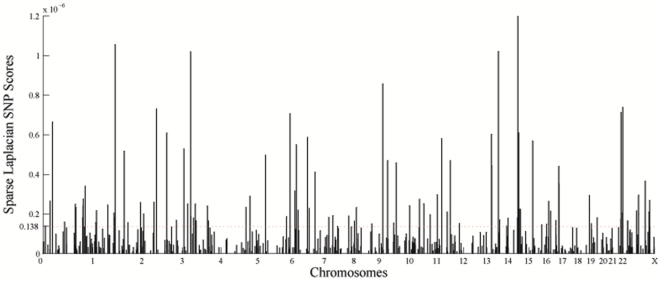
The top 500 ancestral informative SNPs identified by sparse Laplacian approach. The top 100 SNPs are above the dashed red line with scores larger or equal than 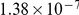.

The distribution of these top 500 informative SNPs in the genome is also provided in **Fig 8**. These markers are relatively uniformly distributed in the genome. Nearby markers are usually redundent in terms of ancestral informativeness because of linkage disequlibrium (LD). The LDs among them are generally small. This pattern suggests that the driving forces that differentiate geographic population structure such as selection, climates, historical events, migration and drift may adapt the whole genome simultaneously rather than a specific region at a time. The top 20 most informative SNPs are provided in Table 2 for interested readers, and the complete set of markers are available upon request.

**Table 2:** Top 20 informative SNPs for seven continental population structure.

Rank	Marker	Chrom	Scores	In
1	rs1834640	15	5.202415e-06	0.4049
2	rs260690	2	1.055257e-06	0.3303
3	rs7143894	14	1.02024e-06	0.2625
4	rs12499585	4	1.018759e-06	0.2242
5	rs4880511	10	8.563433e-07	0.1850
6	rs131026	22	7.385266e-07	0.2345
7	rs6802472	3	7.297752e-07	0.2630
8	rs6001762	22	7.118815e-07	0.2151
9	rs9457490	6	7.062608e-07	0.2042
10	rs2993410	1	6.64077e-07	0.1491
11	rs3751631	15	6.091802e-07	0.2641
12	rs1606871	3	6.0847e-07	0.1400
13	rs3850290	14	6.017834e-07	0.1518
14	rs17207196	7	5.864472e-07	0.2397
15	rs10505879	12	5.80237e-07	0.1510
16	rs8053136	16	5.680701e-07	0.2007
17	rs2390155	7	5.491453e-07	0.1244
18	rs871938	4	5.278809e-07	0.1598
19	rs1348587	2	5.170586e-07	0.1498
20	rs4711546	6	4.970178e-07	0.1772

The top 20 SNPs identified by Sparse Laplacian approach as the most informative markers for global continental population structure and ancestry inference.

## Discussion

The idea of incorporating regularized regression is that majority of the top Laplacian eigenfunction entries are very close to zero and represent random noise rather than true signals of population structure differentiation. The corresponding biological motivation is that some genomic regions undergone evolutionary processes such as selection or historic events more significantly than majority of the genome, though accumulated evidence [25], [26] shows that most of the regions can tell the population diversity. Therefore, suitably forcing small entries of eigenfunctions to be zero with 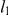 norm can presumably reduce the random effect and improve the entry precision of informative markers.

However, we emphasize that structure informative markers are usually many and the proposed algorithm selects only the most informative ones. The number of selected informative markers with nonvanishing scores increases as the penalty tuning parameter 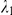 decreases. The rankings of the top most informative markers are quite stable as the tuning parameter varies, which may suffice for most applications. However, other unselected random markers can also detect the underlying population structure except that it generally requires a lot more random markers than those top informative ones. For the selection of tuning parameter, generally there is no universal optimal parameters. For the parameters 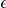 and 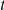 of the undiscovered structures, we usually default 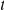 to be 1 and set a large value of 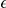 if we are interested in global pattern of the dataset, while setting small values of 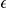 will give more details of the local pattern. Also, the set of informative markers selected by sparse Laplacian approach is less redundant than usual Laplacian regression approach is partly due to the property of LASSO [27] that it tends to select a representative rather than a few from a group of correlated variables, which corresponds to the LD of markers in our setting. While the disadvantage of the LASSO type sparse regression is that it could be time-consuming for hundreds of thousands of markers of thousands of samples.

Generally inclusion of more significant Laplacian eigenfunctions or principal components describing the population structure in our regression setting will improve the overall performance, as the additional eigenfunctions can help locating the specific markers that distinguish the under described subpopulation more efficiently. Here we simply demonstrate that the panel of SNPs selected by our algorithm with K = 2 gives an effective set of informative markers to distinguish seven continents, which is not necessarily optimal. Earlier Lao et al. [28] developed a method based on the informativeness of assignment index 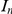 to find markers that differentiate populations and identified 10 SNPs from Y Chromosome Consortium [YCC] panel to successfully differentiate four geographic regions: western Eurasia, East Asia, Africa and America. Their result shows also that there is considerable lack of power when applying the ascertained SNPs to another independent set of population samples. Here we also provide the informativeness of assignment of the top 20 informative markers for interested readers. Needs to mention, addition to the simple application of the 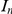 approach on the training dataset. One can also employ suitable clustering algorithms such as STRUCTURE [29] and FRAPPE [30] etc. on the data to infer clusterings of individuals which rather than the predefined individual's membership can then be used to compute 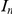.

The incorporated standard SVM with multiple classes feature is not necessarily the optimal approach for the task of multiple continental membership prediction. Even the choice of different kernel functions used can produce slightly different results. Here it is just employed to compare the informativeness between the panel of SNPs selected by PCA and ours. It is possible that other classification techniques such as K-means and variations of SVM etc. may improve the performance. Further investigation in this direction is encouraged. However, we point out that the performance generally depends not only on the number of classes to be predicted but also the variance of each class. The larger the variance is, the more difficult the task is. For the continental membership prediction problem we consider above, the variation within each continent is large since each continent contains quite a few subpopulations with a total 52 worldwide subpopulations. Therefore, it is a challenging task. In the case of population membership prediction for the same number of subpopulations instead of continents or other large geographic regions, the difficulty level drops as the variation of each subpopulation generally is much smaller than that of a continent.

In the current study we exclude the reported related and ambiguous samples [31]. Generally speaking, inclusion of atypical or related samples changes the population structure of the samples. Specifically, atypical samples spread away from major population clusters and related samples cluster toward respective subpopulations. The structure identified by the Laplacian approach is less sensitive to outliers by considering only the *close* neighbors of each individual, compared with PCA. One expects the Laplacian approaches are relatively robust to a set of samples with a small number of related or ambiguous individuals. However, a careful identification of any potential ambiguous or related samples from the genotype data is strongly recommended, as a few softwares such as PREST [32]–[34] are available to achieve such tasks.

In summary, we have developed an algorithm to select population structure informative markers which are also ancestry informative and can be used to recover the original population structure with usually more than 99% genotyping savings. Compared with the PCA approach, the algorithm is not only robust to outliers but also the selected informative markers are less redundant. It is a promising basic tool for the tasks of identifying informative markers and visualization of genetic variation in population genetics and rapidly ongoing genome-wide association studies.

## Materials and Methods

### Data

We use the public global population sample HGDP-CEPH dataset consisting of 1043 individuals from 52 populations of seven geographic continents. All individuals were genotyped using 650K SNP array with total 660,918 SNP markers. We did quality control of the SNPs with the following criteria: minor allele frequency larger than 0.01 and missing rate less than 0.10. After the quality control, 647,483 SNPs are retained. The earlierly reported relatives and ambiguous samples were also excluded in the analysis [31]. The final dataset contains 940 unrelated individuals. The missing genotype data were simply replaced with the average of the nonmissing genotype.

### Basic Notations

Assume there are total 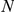 affected and unaffected individuals in the sample. Let 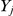 denote the disease status of individual 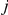, i.e., 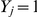 if 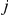 is affected, and 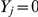 if 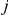 is unaffected. Let 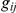 denote the matrix of genotype (0, 0.5, 1) of individual 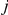 at SNP 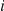, where 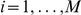. Each SNP 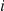 is then normalized by subtracting off the row mean 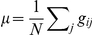, and then divide each entry by 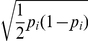, where 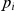 is a posterior estimate of the allele frequency at SNP 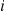 given by 
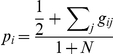
, all missing entries are excluded from the computation. Let's use 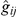 denote the normalized genotype matrix of size 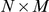, then 
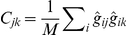
 denotes the standard sample correlation coefficient between individuals 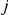 and 
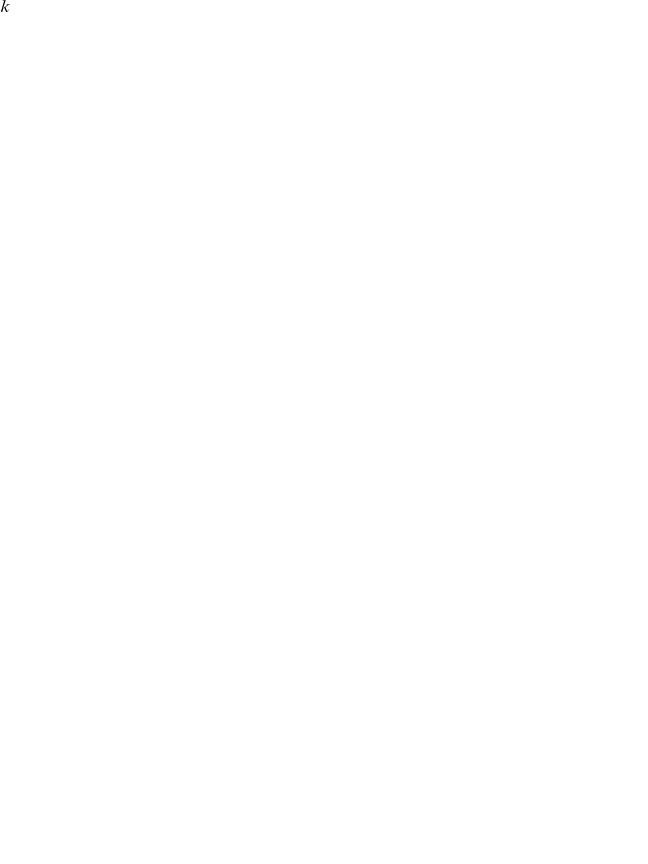
.

### Laplacian of weight matrix

Next we summarize the main ingredients of the recent work [17], [18] on describing population structure using Laplacian eigenfunctions. For each pair of individuals 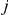 and 
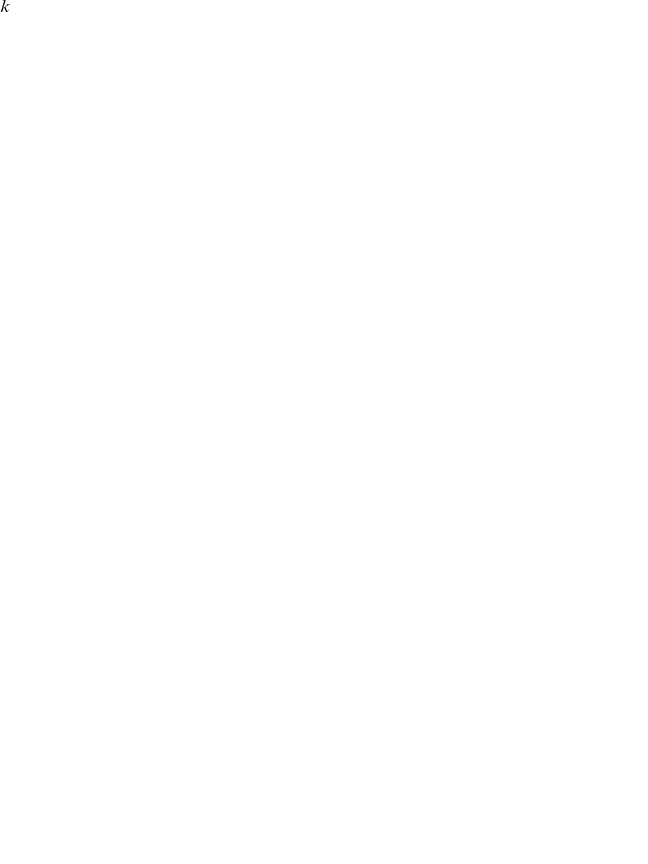
 we assign a distance 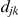 and weight 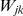. Here we set 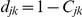. The weight is set to 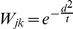 if 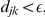 i.e., 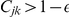, and 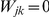 otherwise, where both 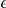 and 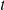 are some preselected positive real numbers. The 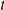 stands for global diffusion scale and in all the computation within the paper we set 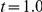. The 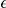 measures the size of each subject's neighborhood in terms of the metric 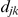. The motivation of the proposed weight is that one counts only pairs who are genetically *close*. The selected Gaussian weight is optimal in certain sense, and it has deep connection with heat kernel on a manifold which gives the general solution to heat equation.

Let 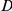 be a diagonal matrix of size 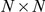 with row sums of 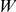 as entries 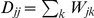. The Laplacian matrix of the weight 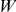 is defined to be 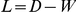. Note that 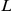 is a symmetric and positive semi-definite matrix. We restrict to the normalized version 
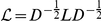
 which is also symmetric. We remark that an alternative normalized version of 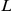 is given by 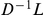, which is not symmetric and can be regarded as a Markov matrix on the graph since each row sum equals one. These two normalizations of Laplacian share the same spectrum [35].

### Laplacian eigenfunctions with sparse loadings

Let 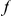 be a function on the graph with value 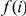 on the 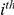 vertex. Then the inner product can be written as 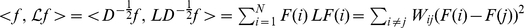, where 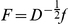 is the normalized version. The eigenfunctions of 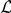, denoted as 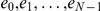 in the increasing order of eigenvalues, are the functions that minimize the weighted variation. That is, 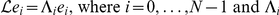 is the 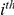 associated eigenvalue. Note 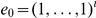 is a trivial solution with equal value on every vertex. The top eigenfunctions of 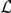 has been recently used to describe population structures [16]–[18].

Next let 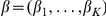 be a matrix of size 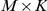, where each column 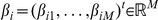 is a unit vector and 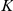 is the number of significant top Laplacian eigenfunctions that one uses to represent the meaningful population structure. We consider the optimization problem below
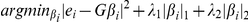
Here 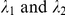 are two nonnegative real numbers which serve as the tuning parameters of the regularized terms 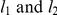 norms of 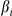. The 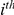 entry 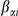 of the loading 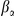 measures the projected signal of the 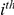 marker on the 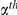 Laplacian eigenvector. It is a general belief that the SNPs that are most informative about the population structure are only a few. That is, the loadings of the eigenvectors are sparse. The 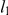 norm term serves as penalty for being nonzero and forces majority of the SNPs with small effect or just random noise to have zero loadings for the corresponding eigenvectors. Linear regression with 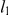 constraint was first introduced as LASSO to the statistical community by Tibshirani [36]. Later 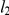 term was also included in order to have the grouping property for variables sharing group effect, for details see Zou et al [37]. Nowadays sparse regression has been applied in many fields such as compressed sensing and gene expression profiles [38]–[40] and various combinations of penalization terms have been proposed in the literature. In the computation we simply set 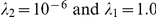. However, one can choose different values.

For the 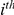 marker, we define a rank statistic 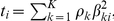 where 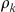
's are weights for each eigenvector. Ideally 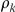 measures the percentage of variance of the data explained by the 
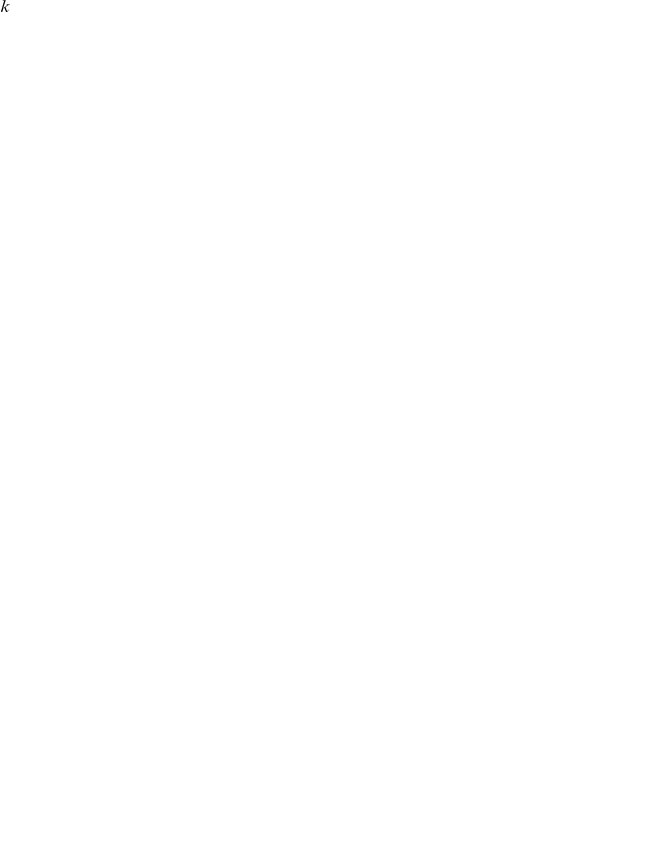
-th eigenvector. A simple alternative statistic is just 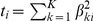 with uniform weights. The markers are ranked in the decreasing order of 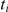
's. The more informative a marker is, the higher it ranks. Majority of the markers have their rank statistic value equal to zero and this reflects the fact that their contribution to the underlying structure is relatively weak.

### Whole Genome Scan

For the computational and memory limitation due to large number of SNPs in whole genome studies, for example in scale of a million SNPs, we propose an alternative stepwise iterative genome scan as follows. In first step, one partitions all the available SNPs randomly into multiple groups whose sizes are around a previously set small number, say, 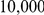. To reduce the effect caused by the linkage disequilibrium (LD) between closeby SNPs, one tries to partition SNPs that are in strong LD into distinct groups. Step two, one applies the proposed selection algorithm to each group and selects a proportion of the top SNPs. Then one merges the selected SNPs into a group and apply the above procedures again.

### Simulation Study

#### A ring species

Following reference [2], an equilibrium population is simulated using the softare MS for population genetics developed by Hudson [41]. The population consists of 100 subpopulations which are equal-spacely arranged on a circle and two isolated subpopulations as outliers. Each subpopulation is assumed to consist of equal number of diploids. During each generation backward in time, a fraction 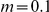 of each subpopulation along the circle is made up of migrants from each adjacent subpopulation and there is no gamete swaps between non-adjacent subpopulations. 10,000 SNP loci were independently simulated with one segregation site per locus and ten individuals were sampled from each subpopulation with total 1020 samples.

### URL

R code for computing Sparse Laplacian Eigenfunctions is available at http://galton.uchicago.edu/~junzhang/LAPSTRUCT.html.
